# Adherence to Mediterranean diet among Lithuanian and Croatian students during COVID-19 pandemic and its health behavior correlates

**DOI:** 10.3389/fpubh.2022.1000161

**Published:** 2022-09-16

**Authors:** Brigita Mieziene, Greta Burkaite, Arunas Emeljanovas, Ilona Tilindiene, Dario Novak, Ichiro Kawachi

**Affiliations:** ^1^Department of Physical and Social Education, Lithuanian Sports University, Kaunas, Lithuania; ^2^Department of General and Applied Kinesiology, University of Zagreb, Zagreb, Croatia; ^3^Department of Social and Behavioral Sciences, Harvard T. H. Chan School of Public Health, Boston, MA, United States

**Keywords:** Mediterranean diet, physical activity, smoking, university students, health-related behaviors

## Abstract

Maintaining healthy behavior, especially in times of crisis like the COVID-19 pandemic, is particularly important for staying healthy. Nutrition is an everyday behavior and along with other health-related behaviors is associated with many health outcomes. The aim of this study was to assess and compare adherence to the Mediterranean diet (MedDiet) and particular food choices among the Mediterranean and non-Mediterranean populations of university students and identify its lifestyle correlates at the outburst of the COVID-19 pandemic. In total, self-reported data on health-related behavior and sociodemographic characteristics were collected from 1,388 study participants, 66.4% were Lithuanians, and 33.6% were Croatians. Results revealed that vegetables, olive oil, fruits, nuts, legumes, and fish were remarkably underconsumed among university students in the Mediterranean and non-Mediterranean countries during the COVID-19 pandemic, and the composite diet is similar between countries. The higher adherence to MedDiet is associated with physical activity (β = 0.15) and non-smoking (β = 0.08). In times of crisis, public health entities should provide knowledge, skills, and tools for healthy nutrition specifying them by age and subpopulation. Interventions at the university should be implemented to build infrastructure and provide an access to health behavior-friendly environments.

## Introduction

The beneficial effect of the Mediterranean diet (MedDiet) on health has been empirically confirmed by many cross-sectional, longitudinal, and experimental studies. Reviews and empirical studies report the benefits of MedDiet in preventing numerous chronic conditions, preterm mortality, and age-related cognitive dysfunction (1–3). Traditionally, MedDiet is related to traditional areas of olive cultivation in the Mediterranean region and is characterized by a high intake of plant and unprocessed foods (1). By contrast, the westernized diet, characterized by a high intake of refined grains, processed meats, animal fats, and high intake of sugar, is related to the increased prevalence of non-communicable diseases, by adversely affecting both gut microbiota and the immune system (4). Meanwhile, a recent study report that poor dietary habits are responsible for more deaths than any other risks globally, including tobacco smoking (5).

The nutritional and health value of separate items in MedDiet are well established. For instance, unsaturated fats (fish, almonds, olive oil) improves blood cholesterol levels, ease inflammation, and stabilize heart rhythms (6). Additionally, fruits and vegetables high in fibers, antioxidants, and minerals prevent the development of some forms of malignancies and degenerative diseases (5). Moreover, intake of foods high in protein (chicken meat, eggs, almonds, Greek yogurt) is related to muscle gain and lower blood pressure (5). The regular consumption of nuts protects cognitive function from deterioration in adults of different ages (7). Here, to note, in recent years, the synergistic effects of the components and the overall combination of healthy foods are in the research loop as it provides a complex perspective on nutrition.

Apart from the benefits of MedDiet to individual and public health, its sustainability is also an issue. For instance, animal products (dairy, egg, meat, and fish) contribute to more than half of the impact on greenhouse gases (GHG) emissions and energy requirements. Meat products are the strongest contributors to GHG emissions and freshwater and dairy products to energy use (8). Meanwhile, plant foods even when processed, in comparison with animal-based foods, have lower GHG emissions and freshwater use (1), which preserve both human and environmental health. Besides its eco-friendly and health effect, MedDiet is also relatively affordable.

In addition to the abovementioned benefits, the MedDiet, being rich in antioxidants, having anti-inflammatory, and potential antimicrobial and immunomodulatory effect is a promising dietary approach to attenuate the severity of COVID-19 infection (9). There were already many arguments in the previous studies that adherence to MedDiet in Mediterranean countries has declined with every generation due to westernization and globalization processes (10). The call for the implementation of MedDiet is also sound globally, across non-Mediterranean and Mediterranean regions (11). However, still in general, the adherence in Mediterranean countries to MedDiet was higher than in other regions (12), especially among older populations (13).

The COVID-19 pandemic brought changes in peoples' lifestyles. Studies conducted during the pandemic found that dietary behaviors have changed, both individually and globally. Younger people (<30 years old) and more educated populations reported higher adherence to the MedDiet compared to the older population and those having lower education after the pandemic started (9). During confinement from shelter-in-place restrictions, people reported lower consumption of sugar-sweetened/carbonated beverages and alcohol. The pandemic limited options for eating out. Meanwhile, the frequency of cooking at home during the confinement was related to greater adherence to the MedDiet (14). Still, results vary in different populations. During COVID-19, the nutrient and caloric intakes decreased in Canadian university students (15). University studies become a new era of a person's living with many lifestyle changes as well. The transition from high school to university can be challenging as might be associated with the development of new health-related habits, and attitudes toward health behavior.

The combination of a healthy diet with physical activity makes the MedDiet a sustainable lifestyle model (16) that can be easily adopted by all population groups of various cultures with country-specific variations (17). However, along with the deteriorating diet, during the confinement, physical activity also decreased (18) across different age groups (15, 19, 20) and sedentary behavior increased (15).

This study is aimed to assess and compare adherence to MedDiet and particular food choice among the Mediterranean and non-Mediterranean populations of university students and identify its lifestyle correlates at the outburst of the COVID-19 pandemic.

Based on the previous research on MedDiet, its adherence across the Mediterranean and non-Mediterranean regions, and health behavior change during the COVID-19 pandemic in this study is hypothesized that students in Lithuania and Croatia have similar MedDiet adherence and the higher adherence is related to higher physical activity, non- or moderate consumption of alcohol, and non-smoking.

## Materials and methods

### Study design and procedure

This study is a part of a bigger study. Study participants in this cross-sectional study were enrolled using snowball sampling—a non-probability, convenience sample gathered in Lithuania and Croatia. Several researchers in both countries formed their initial samples selected from available participants (personal and professional contacts: university students, college students, members of youth organizations, representatives of professional societies, followers, and groups in social networks). Then, these participants were asked to enroll more participants in the study and share the survey's internet link with their friends, and colleagues–potential participants aged 18–36-year-old. An online questionnaire was shared through popular social networks and emails within the period October 2020 to May 2021. The study procedure took ~15 min. For this particular research, data only from university students were extracted.

### Participants

In total for both countries, data were collected from 1,388 study participants, 924 (66.4%) were Lithuanians and 467 (34.6%) were Croatians. Most of each national sample consisted of female participants, 62.0 and 63.6%, among Lithuanians and Croatians, respectively. Informed consent was provided along with the study questionnaire. All participants were informed about the goals of the study, the anonymity of their participation, and the option to stop participation at any time of their filling out the study questionnaire. Respondents agreed to participate in the study by submitting their filled online questionnaire. The study was conducted following the Declaration of Helsinki, and the protocol was approved by the Lithuanian Sports University Ethics Committee (No. SMTEK-50).

### Measurements

#### Dietary pattern

Adherence to a dietary pattern was evaluated using the Mediterranean Diet Adherence Screener (MEDAS) (21), which was previously validated in adult populations in the other Mediterranean and non-Mediterranean counties (21, 22), and used in the Lithuanian (23, 24) and Croatian (14) samples of young adults. The 14 items in the MEDAS scale are included. Two of them represent nutrition habits such as the use of olive oil and the preference for white vs. red meat. The other 12 items cover the frequency or amount of consumption of both healthy (olive oil, vegetables and fruits, fish, nuts, and dishes with homemade sauce) and unhealthy (animal fat, commercial pastries, sugar-sweetened beverages) food items. Following the thresholds distinguishing predefined goals for the health-related consumption of specific food items (25), each item was scored as 0 (does not meet the healthy eating criteria) or 1 (meets the healthy eating criteria). The total score was calculated by summing all item scores. The total score on the MEDAS scale for some calculations was categorized into three categories: ≤7 indicated low adherence, 8–9 indicated medium adherence, and ≥10 indicated high adherence to the Mediterranean diet (21).

#### Physical activity

International Physical Activity Questionnaire (IPAQ) short form (26) was used to evaluate physical activity. However, considering the WHO definition of health-enhancing physical activity for this study only summed minutes spent in moderate and vigorous physical activity (MVPA) per week were used. The threshold of 300 min, following the WHO's latest recommendations for adult PA, was used to distinguish participants into those meeting health-related physical activity requirements (MVPA for ≥300 min/week) and those not meeting health-related physical activity requirements (<300 min/week) (27).

#### Alcohol consumption

Alcohol use was evaluated by asking two questions. One identified if a participant drinks alcohol in general: “Do you drink any alcohol at all?” with answers “Yes” and “No.” Another question identified the frequency of risky drinking per drinking occasion “How often do you have six or more drinks on one occasion?”. Those who identified that they drink that much at least monthly were categorized as heavy drinkers based on the World Health Organization's Alcohol Use Disorders Identification Test (AUDIT), interpretation of item 3 (28). Those who identified that they do not drink at all or drink less than is recognized as heavy drinking were categorized in a group of non- and moderate drinkers.

#### Smoking

Smoking was evaluated by asking participants if they smoke at all with the given answers “Yes” or “No.” Based on the 2014 Surgeon General's Report (29) that there is no safe amount of tobacco use, two categories of smoking were created: smokers and non-smokers.

#### Covariates

Place of residence was indicated by respondents designating themselves as living in a city or a region. Cohabitation was identified by asking “Whom do you live together with?” by choosing between answers “Alone,” “With partner,” “With parents,” and “With roommates.” Further, this indicator was binarized as “Alone” and “Not alone.” Financial status was evaluated by participants denoting their financial status as lower, the same, or higher than average in their country by answering the question “How would you evaluate your (or your family's) financial situation?”. Education was evaluated by asking to indicate the highest achieved degree at the moment of the survey with answers 1–“High school,” 2–“Vocational,” 3–“Higher non-university degree,” and 4–“Higher university degree.” For age, the number of full years was the indicator. Gender was also considered a covariate. Participants had to choose between two categories of their biological gender–men (1) or women (2). Participants also had to indicate their nationality as an open question.

### Statistical analysis

Data were analyzed using SPSS 28.0 software (SPSS Inc., Chicago, IL, USA). Descriptive statistics for determining the means, standard deviations, and frequency distributions of variables used in the study. The chi-square test was employed to identify relationships between nominal and categorical study variables. The prediction of adherence to the Mediterranean diet was identified using hierarchical linear regression analysis. Skewness and Kurtosis of standardized residuals in the regression analysis were in the range between −1 and 1. Student *t* criteria were used for the comparison of the mean difference. Statistical significance was set at a *p* < 0.05.

STROBE Statement—checklist guidelines were followed in organizing this paper.

## Results

In Table 1, results show that distribution between genders is similar between nationalities. Lithuanians and Croatians are of similar age as well. Most students indicate the average financial status and mostly live in a city in both countries similarly. More Lithuanian students live alone or with a partner, instead, Croatian students more often live with parents.

**Table 1:** Sociodemographic and health-behavior characteristics of the study sample.

**Study variable**	**Lithuanians % or mean (SD)**	**Croatians % or mean (SD)**
**Gender**		
Male	38.0	36.4
Female	62.0	63.6
**Financial status**
Lower than the national average	8.4	5.2
The same as the national average	62.5	64.1
Better than the national average	29.2	30.7
**Cohabitation**		
Alone	18.6^***^	8.6
With a partner/spouse	25.2^***^	9.6
With parents	36.7	64.2^***^
With roommates	19.5	17.6
**Place of living**
City	71.7	75.4
Region	28.4	24.6
**Age**	21.08 (2.55)	20.84 (2.12)
**Adherence to MD**		
Mean score	5.16 (1.91)	5.16 (1.94)
Poor	59.7	58.6
Average	39.0	39.4
Healthy	1.4	1.9
**Physical activity**		
Not sufficient	68.3	82.2^***^
Sufficient	31.7	17.8
**Alcohol consumption**		
Risky	11.1	21.9^***^
Moderate or none	88.9	78.1
**Smoking**		
Smoke	21.1	20.3
Do not smoke	78.9	79.7

^*^Significance χ^2^ when ^***^ *p* < 0.001.

The comparison of adherence to MedDiet between Lithuanian and Croatian students (Table 1) did not indicate any statistical differences. In both countries, more than half of students reported poor adherence. Around 4 out of 10 reported average adherence. Only a small percent (1.4% of Lithuanians and 1.9% of Croatians) complied with MedDiet's recommendations. Lithuanian students were more physically active during the COVID-19 pandemic as there were almost one-third of sufficiently physically active Lithuanians and <20% of Croatian students. One out of five students smokes in both countries, and there is no difference between them. However, among Croatians, risky drinking is more prevalent among students than among Lithuanians.

Comparison between nationalities across food items presented in Table 2 revealed inconsistent results. A little bit more than half of the young adults living in Croatia–part of the Mediterranean region, are meeting the recommendation to include olive oil as the main fat in their diet in contrast with one-third of Lithuanians. However, only around a quarter of young Croatians meet the recommended daily amount of olive oil, but still more than Lithuanians (13.1%). More Croatians than Lithuanians consume wine, however, only around 1.9% of Croatians and 0.4% of Lithuanians meet the suggested amount of 7 glasses a week (one glass per day). Almost a quarter of Lithuanians consume a sufficient amount of nuts per week, which is significantly less than eat their peers in Croatia (31.8%). White meat over red is preferred among the majority of university students in both countries; however, there is still a significant difference of 5% in favor of Croatia. The consumption of four out of 14 items in the Mediterranean diet items list does not differ between countries in the Mediterranean region and Lithuania. In particular, in both countries, less than one-third of students consume the recommended 3 fruits a day, without any significant difference. Legumes are underconsumed in both countries, and only 12% of young adults in Lithuania vs. 14% in Croatia include them in their menu. As well, there is no difference in fish and seafood consumption; however, only about one out of ten students consumes a sufficient amount of fish and seafood. Moreover, Lithuanians outperform Croatians in several food items in the list of the Mediterranean diet. In particular, more than half of Lithuanians and Croatians meet the criteria of recommended daily amount of vegetables, with significant favor to Lithuanians. A little bit more than half of both countries' students refuse consumption of red and processed meat on their menu with a slightly significant difference between countries in favor of Lithuanians. There is a difference in consumption of animal fat, more students in Lithuania (40.1%) than in Croatia (31.6%) skip animal fat from their menu. About a half of university students limit their sugary drinks in both countries, by 5% more often in Lithuania. Lithuanians also resist the temptation of pastries more often than Croatians as 76% of Lithuanians vs. 56% of Croatians meet the recommendation to avoid pastries. Most Lithuanians and Croatians (>80%) eat homemade food daily with a slight advantage of 3% among Lithuanians (Table 2).

**Table 2:** The consumption of different food items among Lithuanians and Croatians university students.

**Nutrition item meeting recommendations**	**Lithuanians (%); χ^2^**	**Croatians (%); χ^2^**
Olive oil as main culinary fat (yes)	33.5	52.7^***^
Amount of olive oil/day (≥4 tbsp)	13.1	25.3^***^
Servings of vegetable/day (≥2 servings)	59.9^***^	49.9
Fruits units/day (≥3)	27.9	31.5
Servings of red meat, hamburger, meat products/day (<1)	56.9^*^	51.9
Servings of butter, margarine, cream/day (<1)	40.1^***^	31.6
Sweet or carbonated beverages/day (<1)	55.0^*^	49.7
Glasses of wine/wk (≥7 glasses)	0.4	1.9^**^
Servings of legumes/week (≥3)	12.1	14.3
Servings of fish or shellfish/wk (≥3)	10.2	7.5
Commercial sweets or pastries, times/wk (<3)	76.8^***^	54.7
Servings of nuts/week (≥3)	26.1	31.8^**^
White meat instead of red or processed meat (yes)	76.0	80.9^*^
Homemade vegetables, pasta, rice and other food/wk (≥2)	87.1	84.3

^*^Significance χ^2^ when ^*^ *p* < 0.05; ^**^ *p* < 0.01; ^***^ *p* < 0.001.

Results of multiple regression analyses show (Table 3) that among sociodemographic indicators in the first model, being women, older age is associated with higher adherence to the MedDiet. Neither financial status nor cohabitation, place of residence, or nationality predicted better adherence to MD. Among health-related behavior indicators, meeting physical activity recommendations and non-smoking is related to higher students' adherence to MedDiet.

**Table 3:** Prediction of Mediterranean diet from sociodemographic, health behavior variables in Lithuanian and Croatian university students.

**Variable**	**Standardized beta**	
	**Model 1**	**Model 2**
Gender (female)	0.076^**^	0.113^***^
Age	0.097^**^	0.099^**^
Financial status	0.048	0.052
Cohabitation (not alone)	−0.054	−0.054
Place of residence (region)	−0.026	−0.029
Nationality (CRO)	−0.014	0.023
PA (Sufficiently active)		0.150^***^
Alcohol intake (non and moderate drinkers)		0.039
Smoking (non-smokers)		0.081^**^
ΔR	0.017^***^	0.045^***^

LT, Lithuanians; CRO, Croatians; ^*^Significance χ^2^ when ^**^ *p* < 0.01; ^***^ *p* < 0.001.

## Discussion

This study sought to assess and compare adherence to MedDiet at the beginning of the COVID-19 pandemic between Lithuanian and Croatian university students and to identify the links of adherence to MedDiet to other health behaviors and sociodemographics within the Mediterranean and non-Mediterranean populations.

The results indicated that in terms of the composite score of the MEDAS scale no statistical difference was indicated. More than half of university students have poor eating habits in both countries, despite the traditional peculiarities in nutrition. Around 40% have average adherence and only 1.4 and 1.9%, in Lithuania and Croatia, respectively, have good adherence to MedDiet. A systematic literature review revealed that Mediterranean populations have been showing moderate adherence to MedDiet in the past 10 years (30). Regardless of the expansion of Mediterranean traditions of nutrition around the globe, in Lithuania, the deterioration is observed moving from adolescence to adulthood and from pre-pandemic to pandemic situations. Previous studies in Lithuania revealed that there is around 14% of adolescents are meeting the criteria of healthy nutrition (31). The number is reduced by half among young adults when only 7% of those meeting recommendations on the MEDAS scale were observed in the pre-pandemic study (23). Similarly, in Croatia, the comparative study indicated that the percentage of good adherence to MedDiet is lower among older age groups of youth and is about 13% among students (measured with the KIDMED scale) (32). So, the results of this study show an even worse reduction. This led to the premise that the COVID-19 pandemic in short term might have deteriorated the eating habits of youth in Lithuania and Croatia. However, there is a different opinion in the scientific literature, which states that young people during confinement had more free time to cook homemade meals and thus, comply with healthy eating standards. For instance, a study in Spain found that adherence to the MedDiet increased significantly by 0.8 points during the confinement. However, this increase refers to the first 3 weeks of confinement (33) when people focused on their household chores to use their free time, as going out was restricted, and studies and work online were not then well-established. Although some studies point out that people increased their cooking frequency during the confinement, which was associated with an increase in vegetables, legumes, and fish, as well as seafood consumption (14), other studies also noticed a higher frequency of snaking and by 50% increased demand for confectionery products (34), which might deprive the benefits of the homemade food. Other studies found that during the COVID-19 pandemic the quality of students' diet was poorer. In particular, they consumed lower amounts of grains, fruits, vegetables, dairy, and nuts (15).

The review of other studies on adherence to MedDiet during the COVID-19 pandemic indicated that most people have not changed their eating patterns; however, there were more of those who increased than decreased their adherence. Most studies found that people increased their consumption of vegetables, fruits, nuts, olive oil, and dairy products compared with the previous studies (35). One other comparative study across 16 Mediterranean and non-Mediterranean countries found higher adherence in countries identifying that there are 4.58% in Lithuania and 10.1% in Croatia of those with high adherence to MedDiet during the confinement. However, the mean scores in the Lithuanian sample in that study and the current study were very similar like 5.16 in this study and 5.13 in Molina-Montes et al. study (36) at the moment of confinement. However, their study included all adults with no age limit, so probably, the higher frequency of high adherence could be explained by the older age of their participants as most of the participants were 51 years of age or older and only <40% of their study sample were in the same age group as our sample. Another study in Croatia also confirms that high adherence to MedDiet is particularly low among younger participants compared to the older ones (37). Younger generations in the Mediterranean area are more prone to the “westernization” process, which also affects their food habits toward palatability and sensory perception–driven food, and have increasingly similar patterns of food availability (mainly non-Mediterranean food groups) among the Mediterranean and non-Mediterranean areas (38), while older generations stick with their traditional meals and food preferences (39). Lithuania has the smallest group of people with high adherence to MedDiet among 16 countries in the Southern Mediterranean region, Balkan Mediterranean region, and non-Mediterranean region (European countries). The highest percentage of high adherence was among Mediterranean countries, especially in Portugal (27%) and Spain (23%) (36). The Molina-Montes and colleagues' survey was administered at the beginning of the first confinement, meanwhile, ours is already in the Autumn 2020–Winter 2021 period.

In this study, university students in both countries mostly comply with the recommendation to choose white meat over red one (76 and 81% in Lithuania and Croatia, respectively) and eating at homemade dishes with sofrito (88 and 84% in Lithuania and Croatia, respectively). Fewer Lithuanians than Croatians consume commercial pastries. Some authors point out that the demand for confectionery products and butter also increased in more than 50% of the population during the confinement in another Mediterranean country–Spain (34). Underconsumption of olive oil, although lower among Lithuanians, is observed in both countries as only 13 and 25% of Lithuanians and Croatians, respectively, meet the recommendation for the amount of olive oil per day. For instance, among Lebanese students, there was observed a relatively high spread of usage of olive oil in cooking (86.3%), and 50.3% of them consume a recommended amount of olive oil per day (40). Underconsumption of fruits, nuts, fish, and legumes is also spread in both countries as less than one-third of young adults meet the recommended amounts. Another study revealed that although 30% of students showed high adherence to the MedDiet, only 42% of the participants had a high consumption of vegetables, which are lower than in our study, and 85% a low consumption of energy drinks, which is higher than in our study. The consumption of fruits and vegetables in that study was also related to higher psychological adjustment and health perception (41). Given the benefits that fruits and vegetables have on cognitive function, while nuts and olive oil have an impact on cardiovascular health and mortality (1), the future health of students raises concerns. Moreover, switching diet toward the plant-based is contributing not only to health but also to a healthy environment, which in turn again contributes to individual and public health. Empirical evidence confirms that MedDiet is a part of a sustainable environment (8). Some authors based on empirical research suggest that poor nutrition literacy leads to the consumption of more food associated with the Western diet (fried food, sugar-sweetened beverages, red meat, and processed food), while good nutrition literacy is associated with Mediterranean diets (vegetables, olive oil, and nuts) (42). Policies making MedDiet more animal-based diets less affordable are encouraged.

Moreover, the results of this study show that healthier nutrition for students is also related to other health behaviors, in particular, higher physical activity and non-smoking. Seems like some students just have a healthy person profile, where health-related behaviors are clustered. Other studies similarly confirm that smokers have lower adherence to MedDiet (43–45). Better adherence to MedDiet was related to higher physical activity among Lithuanian adolescents (24) and adults (23) in previous studies. Specifically, those who consume more olive oil, vegetables, fruits, legumes, fish, and nuts are more physically active (23). Similarly, adherence to MedDiet was lower in current smokers and in those who spent more time watching TV and higher in those who were more physically active, among the elderly in Spain (46).

Further, better adherence to MedDiet is related to the female gender and students' older age. The age's links to better MedDiet were discussed above. Also, older students' age means more advanced education, which in turn might affect health-related behavior. Meanwhile, gender-related links to MedDiet are inconsistent in other studies. Similarly, in Maltese adults being women, non-smoker, and having older age was associated with higher adherence to the Mediterranean diet (44). Men were less likely to show good adherence among Croatians in one of the previous studies (37). These results might suggest the premise that older age, especially for women means more time spent cooking and eating at home, which is related to higher adherence (34). Another study found that women pay more attention to nutrition quality than males, perceive fewer barriers to food price when it comes to choosing food, and have a higher perception of the benefits of diet quality (47). So, women probably have a more conscious approach to what they eat than males, while in a country like Lithuania the food choice in favor of MedDiet still must be made consciously compared with Mediterranean countries where availability of MedDiet is higher and have long traditions.

### Limitations

Some limitations should also be acknowledged. Selection bias was possible due to the snowball sampling, e.g., people who had a poor diet encouraged their friends (who also had a poor diet) to join the study. However, some other abovementioned studies in similar populations also present results that do not dramatically differ from this study's results. Another limitation is the cross-sectional nature of the study. We were not able to compare the switch in diet from pre- to pandemic. Instead, we examined nutrition habits when the acute phase of the pandemic was over, in Autumn 2020–Winter 2021, and expectedly dietary patterns became more or less stable in the new reality.

## Conclusions

Vegetables, olive oil, fruits, nuts, legumes, and fish were remarkably underconsumed among students in Lithuania and Croatia during the COVID-19 pandemic. Mediterranean students in Croatia prefer and consume more olive oil, nuts, drink wine, and choose white meat over red more often than non-Mediterranean Lithuanian students, while Lithuanians consume vegetables more often. Also, non-healthy Western diet-related foods were more common among Croatian students. However, the composite score of diet, which is more important for health as it considers compensatory effects of single food items, is similar between both countries. Campaigns like fruits instead of sweets or fish instead of red meat should be incorporated into organizational (like universities or companies) health-enhancing strategies and the availability of healthy foods provided. Along, availability of unhealthy foods should be restricted. Health policy restricting unhealthy food availability should encourage food providers at any level to present more healthy foods for the market in general and to the university campus in particular. In times of crisis, or social isolation and not only then but public health entities should also provide knowledge, skills, and tools for healthy nutrition specifying them by subpopulations and including the university student population. Health organizations, scientists, food companies, and IT specialists could collaborate to create smart tools to enhance healthy nutrition among students who are usually the main users of innovative technologies. Healthy nutrition, among other health behaviors, should be also emphasized in university studies. Interventions at the university should be implemented to build infrastructure and provide an access to health behavior-friendly environments.

## Data availability statement

The raw data supporting the conclusions of this article will be made available by the authors, without undue reservation.

## Ethics statement

The study was approved by the Institutional Ethics Committee of Lithuanian Sports University (protocol code no. SMTEK-50, 29/09/2020). The patients/participants provided their written informed consent to participate in this study.

## Author contributions

BM and DN: conceptualization. BM and AE: methodology. BM: software, formal analysis, data curation, and funding acquisition. IK and DN: validation and writing—review and editing. IT and DN: investigation. GB: resources. BM and GB: writing—original draft preparation. IT: visualization. AE: supervision. All authors have read and agreed with the published version of the manuscript.

## Funding

This research has received funding from European Regional Development Fund (grant number 09.3.3-LMT-K-712-19-0015) under grant agreement with the Research Council of Lithuania. The funders had no role in the design of the study, in the collection, analyses, interpretation of data, writing of the manuscript, and the decision to publish the results.

## Conflict of interest

The authors declare that the research was conducted in the absence of any commercial or financial relationships that could be construed as a potential conflict of interest.

## Publisher's note

All claims expressed in this article are solely those of the authors and do not necessarily represent those of their affiliated organizations, or those of the publisher, the editors and the reviewers. Any product that may be evaluated in this article, or claim that may be made by its manufacturer, is not guaranteed or endorsed by the publisher.
